# Evaluating a clinical tool (FAMCAT) for identifying familial hypercholesterolaemia in primary care: a retrospective cohort study

**DOI:** 10.3399/bjgpopen20X101114

**Published:** 2020-11-18

**Authors:** Ralph K Akyea, Nadeem Qureshi, Joe Kai, Simon de Lusignan, Julian Sherlock, Christopher McGee, Stephen Weng

**Affiliations:** 1 Primary Care Stratified Medicine (PRISM), Division of Primary Care, University of Nottingham, Nottingham, UK; 2 Royal College of General Practitioners (RCGP) Research and Surveillance Centre (RSC), London, UK; 3 Nuffield Department of Primary Care Health Sciences, University of Oxford, Oxford, United Kingdom; 4 Department of Clincial and Experimental Medicine, University of Surrey, Guildford, UK

**Keywords:** familial hypercholesterolaemia, case-finding, FAMCAT, validation, primary health care, general practice, lipid metabolism disorders

## Abstract

**Background:**

Familial hypercholesterolaemia (FH) is an inherited lipid disorder causing premature heart disease, which is severely underdiagnosed. Improving the identification of people with FH in primary care settings would help to reduce avoidable heart attacks and early deaths.

**Aim:**

To evaluate the accuracy of the familial hypercholesterolaemia case ascertainment identifcation tool (FAMCAT) for identifying FH in primary care.

**Design & setting:**

A retrospective cohort study of 1 030 183 patients was undertaken. Data were extracted from the UK Royal College of General Practitioners (RCGP) Research and Surveillance Centre (RSC) database. Patient were aged >16 years.

**Method:**

The FAMCAT algorithm was compared with methods of FH detection recommended by national guidelines (Simon Broome diagnostic criteria, Dutch Lipid Clinic Network [DLCN] Score, and cholesterol levels >99^th^ centile). Discrimination and calibration were assessed by area under the receiver operating curve (AUC) and by comparing observed versus predicted cases.

**Results:**

A total of 1707 patients had a diagnosis of FH. FAMCAT showed a high level of discrimination (AUC = 0.844, 95% confidence interval [CI] = 0.834 to 0.854), performing significantly better than Simon Broome criteria (AUC = 0.730, 95% CI = 0.719 to 0.741), DLCN Score (AUC = 0.766, 95% CI = 0.755 to 0.778), and screening cholesterols >99 th centile (AUC = 0.579, 95% CI = 0.571 to 0.588). Inclusion of premature myocardial infarction (MI) and fitting cholesterol as a continuous variable improved the accuracy of FAMCAT (AUC = 0.894, 95% CI = 0.885 to 0.903).

**Conclusion:**

Better performance of the FAMCAT algorithm, compared with other approaches for case finding of FH in primary care, such as Simon Broome criteria, DLCN criteria or very high cholesterol levels, has been confirmed in a large population cohort.

## How this fits in

Many individuals with FH, an inherited lipid disorder, remain undiagnosed globally. This results in lost opportunities to identify and prevent many cases of premature heart disease and premature death. This study evaluated the accuracy of a clinical tool (FAMCAT) in identifying FH in primary care. In this study, FAMCAT has been confirmed to have a better predictive accuracy compared with other recommended approaches (Simone Broome criteria, DLCN criteria,- and very elevated cholesterol alone) for FH casefinding in primary care.

## Introduction

FH is a common inherited cause of raised cholesterol, affecting up to 320 000 adults in the UK and 834 000 adults in the US (one in 250 prevalence for the adult general population).^1^ Despite internationally recognised guidelines recommending clinicians actively identify individuals in primary care settings,^2–4^ up to 80% of individuals with FH are still not identified,^3,5^ leading to many avoidable heart attacks and early deaths. FH is a condition where preventive interventions to reduce premature cardiovascular disease (CVD), such as high-intensity statins, are highly effective.^6,7^

Current approaches to clinically predict FH-causing mutation in primary care use the Simon Broome diagnostic criteria, DLCN criteria, Make Early Diagnosis to Prevent Early Deaths (MEDPED), or total cholesterol >99^th^ percentile (>7.5 mmol/l aged <30 years; >9.0 mmol/lL aged >30 years).^2,8^ The Simon Broome criteria,^2^ most commonly used in the UK, recommend that individuals with a total cholesterol concentration of >7.5 mmol/l and a family history of premature heart disease should be classified as having probable FH in primary care and should be referred for further lipid specialist assessment. Patients who then also meet specific clinical diagnostic criteria (for example, tendon xanthoma), or diagnosis by genetic testing, are categorised as having definite FH. The DLCN criteria^9^ use a points-based scoring system to classify possible, probable, or definite FH on the basis of differing LDL cholesterol thresholds, family history of premature vascular disease and raised cholesterol, personal history of premature vascular disease, clinical signs such as tendon xanthoma and arcus senilis, or presence of genetic mutation. MEDPED criteria use age-stratified total-cholesterol thresholds for both the general population and relatives, depending on degree of relation.^10^ Identifying patients that fulfil these criteria in primary medical care settings usually leads to further specialist assessment but may be inefficient given only around 25% of referred patients may be subsequently confirmed to have FH.^11,12^ The National Institute for Health and Care Excellence (NICE) recommends assessment against Simon Broome criteria or DLCN criteria to make a clinical diagnosis of FH in primary care settings.^2^

A case-finding algorithm, FAMCAT, has been previously derived and validated using data from almost 3 million primary care patients (including over 5000 cases of FH) from 681 primary care centres in the Clinical Practice Research Datalink (CPRD) database.^13^ The algorithm had a high predictive accuracy to identify patients with documented FH in primary care, with an AUC of 0.86.^13^ AUC is an overall measure of the ability of a test to discriminate whether a specific condition is present or not present.^14^ AUC value lies between 0.5 and 1 where 0.5 denotes a poor accuracy and 1 denotes a perfect accuracy. This study aimed to externally validate the FAMCAT algorithm in the UK’s RCGP RSC database, which is a separate database from the CPRD database from which the algorithm was originally derived.

## Method

### Study design and population

Primary care data were extracted from the RCGP RSC database in the UK (Supplementary Method S1). The RCGP RSC sentinel system is the principal primary care public health surveillance data used by Public Health England for the UK NHS.^15,16^ A retrospective cohort study was undertaken in a large population of primary care patients. This comprised a randomly selected sample of adult patients registered for primary medical care from 1 January 1999, who were followed-up until 31 January 2017. The patients had at least one documented total or low-density lipoprotein (LDL) cholesterol measurement (necessary for establishing a suspected diagnosis). The cohort comprised all patients who were actively registered and contributing data, and had visited their family medical practice up until the end date of when data were extracted. For patients who were diagnosed with FH, the date of diagnosis was specified as their ending date to ensure all predictors remained temporal to their diagnosis.

Patients aged <16 years were excluded from the analysis as cholesterol thresholds for diagnosis and treatment of FH in children differ from adults.^2^ Patients were also excluded if they had a prior FH diagnosis before the study entry date (1 January 1999) or a diagnosis of other inherited lipid disorders.

The starting time point for database interrogation was consistent with the start date used when deriving the FAMCAT algorithm (Supplementary Table S1) using CPRD.^13^

### Outcome

The primary outcome was defined as the incident diagnosis of FH, identified from a patient record, between 1 January 1999 and 31 January 2017. FH is specifically coded in UK primary electronic health records (EHRs) using the internationally recognised Read coding system. This diagnostic code is entered into primary care electronic records after specialist lipid assessment, based on clinical phenotype, and/or by genetic test.

### Predictor variables

FAMCAT was developed as a multivariate logistic regression model, stratified by sex, to calculate an individual’s probability of having FH.^13^ Box 1 summarises all 10 predictors that were incorporated into FAMCAT. Age, cholesterol levels, and triglycerides were categorised. Statin potency was determined using classifications based on a publication by Law *et al*,^17^ incorporated in the most recent UK NICE guidelines on lipid modification (Supplementary Table S2).^18^ Secondary causes of raised cholesterol, such as diabetes and chronic kidney disease, were included as predictor variables for lower probability of FH.

Box 1 **. Summary of predictor variables in FAMCAT**

- Sex (male or female)
- Age in years (16–24; 25–34; 35–44; 45–54; 55–64; 65–74; 75–84)
- Highest cholesterol measurement recorded (mmol/l)
  - Ideal: TC ≤5 or LDL-C ≤3.3
  - High: TC >5 to ≤6.5 or LDL-C >3.3 to ≤4.1
  - Very high: TC >6.5 to ≤7.5 or LDL-C >4.1 to ≤4.9
  - Extremely high: TC >7.5 or LDL-C >4.9
- Triglycerides within 1 month of highest cholesterol measurement (mmol/l)
  - Idea: <1.7
  - Borderline high: ≥1.7 to <2.3
  - High: ≥2.3 to <5.6
  - Very high: ≥5.6
  - Not assessed
- Lipid-lowering drugs prescribed within 1 month of highest cholesterol measurement (none; fibrate, bile acid sequestrant, or nicotinic acid; low-potency statin; medium-potency statin; high potency statin)
- Family history of familial hypercholesterolaemia (no or yes)
- Family history of myocardial infarction (no or yes)
- Family history of raised cholesterol (no or yes)
- Type 1 or type 2 diabetes (no or yes)
- Chronic kidney disease (no or yes)

TC = total cholesterol; LDL-C = low-densitiy lipoprotein cholesterol

### Validation of the FAMCAT algorithm with comparator models

The FAMCAT logistic regression equation developed in the CPRD database was applied directly to every patient in the cohort to calculate each patient’s probability of having FH. This was done by applying the untransformed regression coefficients and constant term (provided in Supplemetary Table S1). Descriptive characteristics of the study population were provided: patient demographics and clinical characteristics. Patients with no data record for any clinical variables, such as diabetes, chronic kidney disease, and prescribing of statins, were considered either to not have the condition or not been prescribed the drug.

Performance of the risk prediction models was assessed by discrimination and calibration.^19^ Specifically, discriminatory accuracy was assessed for all three models using the AUC or Harrell’s *c*-statistics; with higher values representing better discrimination. To generate CIs for the *c*-statistics, a jack-knife procedure^20^ was used to estimate standard errors. The discrimination of FAMCAT was also compared against Simon Broome diagnostic criteria,^2^ DLCN Score,^9^ and a simple classification of total cholesterol >99^th^ centile^2^ (a new recommendation made by the NICE guideline committee in the latest 2019 update) for determining possible FH. Predictors included in the Simon Broome and DLCN criteria were extracted using Read clinical classification codes and applied directly to the cohort.

Calibration was defined as how closely the predicted probability of FH agrees with the expected probability of FH. This was assessed by plotting the observed number of cases of FH against the expected number of cases of FH for each tenth of predicted probability.^21^

### Optimisation of the FAMCAT algorithm

To develop an optimised FAMCAT algorithm, the 10 predictors in the FAMCAT algorithm developed from the CPRD database were considered as *a priori* predictors. History of premature atherosclerotic CVD such as coronary heart disease and peripheral vascular disease (PVD) have been shown to be significantly associated with FH.^22^ These conditions related to FH were explored as potential predictors and, hence, included in the model and discriminatory performance of the model assessed using AUC. These new predictors included a personal history of premature MI and history of PVD. Cholesterol level was built-in as a continuous variable for these optimised models, with an interaction term to specify whether the measurement was a total or LDL-cholesterol.

The study findings are reported in accordance with the Transparent Reporting of a multivariable prediction model for Individual Prognosis Or Diagnosis (TRIPOD) recommendations (Supplementary Table S3).

### Patient involvement

Involvement of patients and relevant advocate groups at all stages of the previous and current related research projects has proved invaluable. This involvement has helped to further focus the study design, output, and dissemination on the needs of the public and the benefits that can be delivered for the community. FH patient representatives for this research project attended study steering meetings to advise on study conception and preparation, funding application, review of study protocols, and have contributed to interpretation, presentation, and dissemination of the findings.

## Results

### Study population

From the 1  031 11 patients identified from the RCGP database, 1228 patients were excluded owing to having other inherited lipid disorder or having all of their cholesterol measurements documented after a diagnosis of FH. The cohort of patients included in the analysis comprised 1 030 183 (52.1% female) eligible patients from 1 January 1999 to 1 September 2017. There were 649 men (0.13%) diagnosed with FH compared to 1058 women (0.2%). The baseline age of the cohort was 56 years (standard deviation [SD] = 15.3) for men and 57 years (SD = 16.7) for women. The mean highest total cholesterol was slightly higher in women at 5.8 mmol/l (SD = 1.3) than in men (5.6 mmol/l; SD = 1.2). Table 1 shows the full details of baseline characteristics for the entire cohort.

**Table 1.** Clinical characteristics for the cohort of patients aged >16 years

	**Men**	**Women**
***Characteristics***		
Total sample size, *n* (%)	493 400 (47.9)	536 783 (52.1)
Diagnosed with familial hypercholesterolaemia, *n* (%)	649 (0.13)	1058 (0.2)
Baseline age, years (SD)	56 (15.3)	57 (16.7)
History of coronary heart disease <60 years, *n* (%)	15 232 (3.1)	8203 (1.5)
***Ethnic group,* n *(%)***		
White	319 439 (64.7)	358 612 (66.8)
Asian	26 916 (5.5)	27 749 (5.2)
Black	14 181 (2.9)	17 484 (3.3)
Mixed	3662 (0.7)	4465 (0.8)
Other	4151 (0.8)	4254 (0.8)
Unknown	125 051 (25.3)	124 219 (23.1)
***Lipid profile, mmol/l (SD)***		
Highest TC recorded	5.6 (1.2)	5.8 (1.3)
High LDL cholesterol recorded	3.4 (1.0)	3.5 (1.1)
Triglycerides during cholesterol measurement	1.6 (1.0)	1.4 (0.8)
***Lipid-lowering drug usage at time of cholesterol measurement*,** n **(%)****		
Prescribed fibrate, bile acid sequestrant, nicotinic acid	1158 (0.2)	1491 (0.3)
Prescribed low-potency statin	6174 (1.3)	5521 (1.0)
Prescribed medium-potency statin	45 510 (9.2)	38 948 (7.3)
Prescribed high-potency statin	21 860 (4.4)	17 183 (3.2)
***Family history*,** n **(%)****		
Family history of FH	1136 (0.2)	1851 (0.3)
Family history of raised cholesterol	6698 (1.4)	10 144 (1.9)
Family history of myocardial infarction	28 213 (5.7)	36 175 (6.7)
***Secondary causes of high cholesterol at time of cholesterol measurement*,** n **(%)****		
Diagnosed with diabetes	84 490 (17.1)	68 978 (12.9)
Diagnosed with chronic kidney disease	53 866 (10.9)	71 332 (13.3)

Asian includes Indian, Pakistani, Bangladeshi, Chinese, and other Asians. Values are numbers and proportions unless stated otherwise

FH familial hypercholesterolaemia LDL low-density lipoprotein SD standard deviation TC total cholesterol

### External validation

#### Discrimination

**Figure 1.**
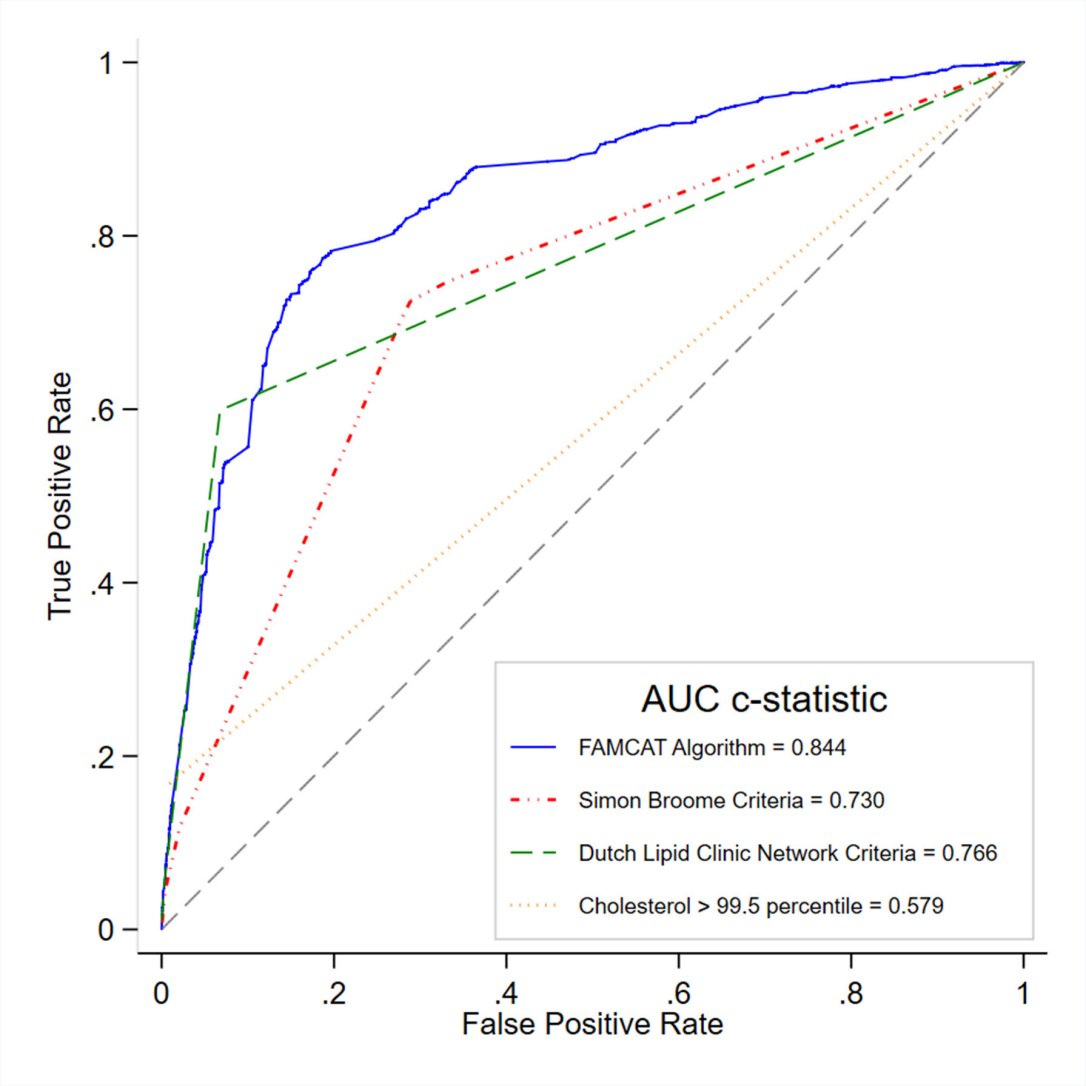
Receiver operating curves derived from the external validation cohort (*n* = 1 030 183) for models of identifying familial hypercholesterolaemia in general practice (FAMCAT discrimination compared with recommended diagnostic criteria). Higher area under the curve (c-statistic) confers better discrimination

Table 2 shows the discrimination of the FAMCAT algorithm compared with other clinical criteria. External validation of the FAMCAT model in the RCGP RSC database showed high level of discrimination (AUC = 0.844, 95% CI = 0.834 to 0.854). The performance of FAMCAT showed significantly better discrimination compared with Simon Broome criteria (AUC = 0.730, 95% CI = 0.719 to 0.741) and DLCN Score (AUC = 0.766, 95% CI = 0.755 to 0.778). Figure 1 shows the receiver operating characteristics curves of the various models.

**Table 2.** Model discrimination in the external validation cohort for identifying familial hypercholesterolaemia in general practice (*n* = 1 030 183)

**Models**	**AUC**(**c-statistic**)	**Standard error** ^a^	**95% CI**
FAMCAT	0.844	0.005	0.834 to 0.854
Simon Broome criteria^b^	0.730	0.006	0.719 to 0.741
Dutch Lipid Clinic Network criteria^c^	0.766	0.006	0.755 to 0.778
Cholesterol >99^th^ centile^d^	0.579	0.005	0.571 to 0.588

^a^Jack-knife procedure to estimate standard errors.^20^ ^b^Total cholesterol >7.5 mmol/l or LDL-cholesterol >4.9 mmol/l+ family history of premature myocardial infarction.^2^ ^c^Score based on LDL-cholesterol, family history, clinical history, and physical examination.^9^ ^d^The UK National Institute for Health and Care Excellence recommendation of screening for FH for cholesterol >99^th^ centile. That is, total cholesterol >9.0 mmol/l or LDL-cholesterol >6.6 mmol/l if aged >30 years; total cholesterol >7.5 mmol/l or LDL-cholesterol >4.9 mmol/l if aged ≤30 years.^2^

#### Calibration

**Figure 2.**
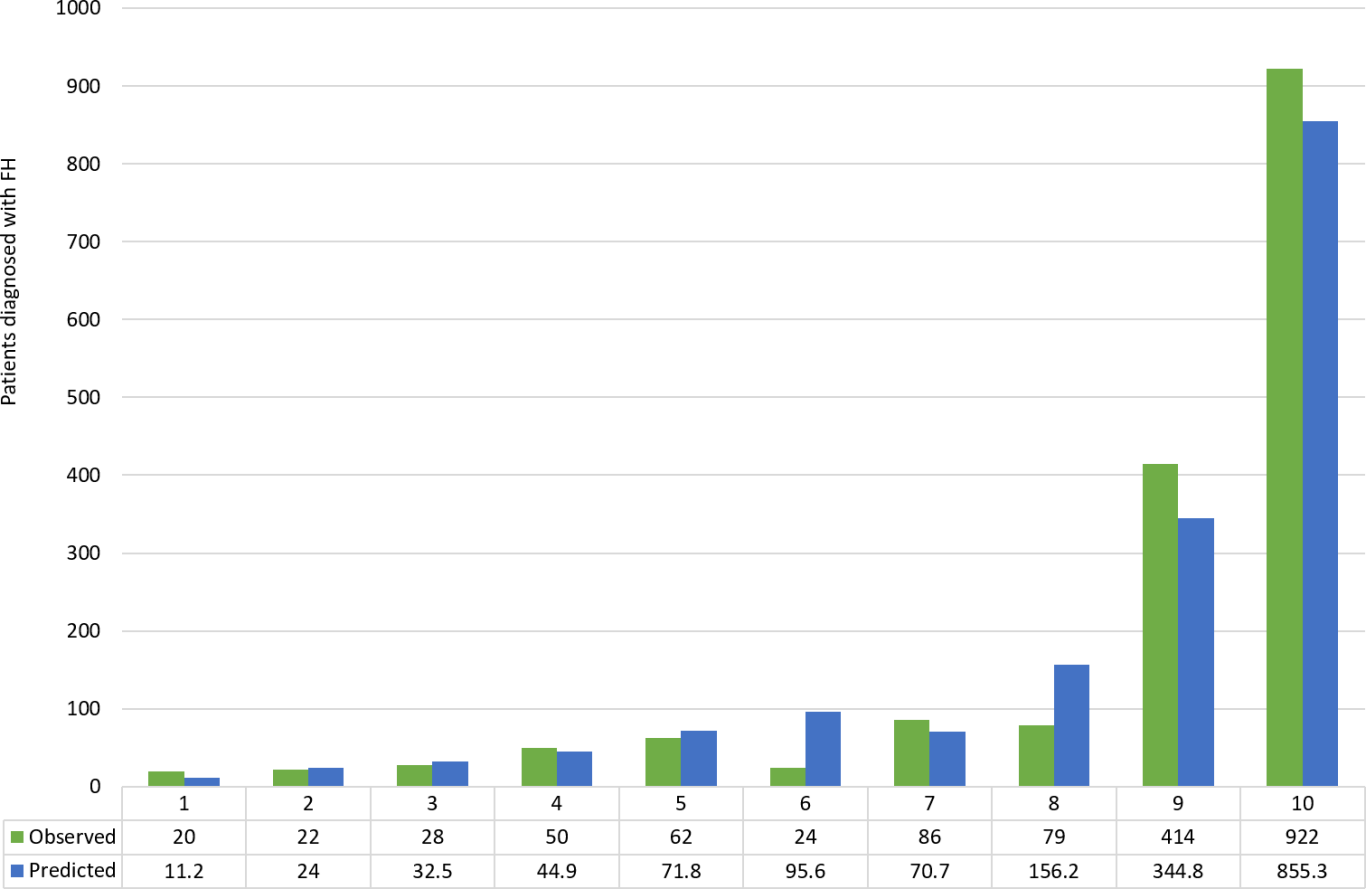
FAMCAT model calibration of observed versus predicted cases of familial hypercholesterolaemia in the external validation cohort by deciles of predicted probability

The model showed good calibration across all deciles between observed and predicted cases, with slight under prediction of cases in the highest two deciles (Figure 2). There was an expected sharp increase in observed and predicted cases in the highest deciles of predicted probability where 414 cases were observed and 344.8 cases were predicted for the 9th decile, and 922 cases observed and 855.3 predicted for the 10^th^ decile.

#### Sensitivity and specificity

A threshold corresponding to the top decile (10^th^) of predicted probability was used for case finding in the primary care setting,^13^ a probability cut-off of ^1^/_250_ or 0.004, the estimated prevalence of FH.^3^ Using this cut-off, FAMCAT achieved a sensitivity of 77.5% (95% CI = 75.4% to 79.5%) and specificity of 81.1% (95% CI = 81.0% to 81.2%) with a corresponding positive predictive value of 0.68% (95% CI = 0.64% to 0.71%) and a negative predictive value of 100%.

### Optimised FAMCAT models

To optimise the FAMCAT model, cholesterol level was fitted as a continuous variable. Predictors considered to be related to FH (that is, personal history of premature MI and personal history of PVD) were included, with the risk factors or variables from FAMCAT algorithm serving as *a priori* predictors.

**Figure 3.**
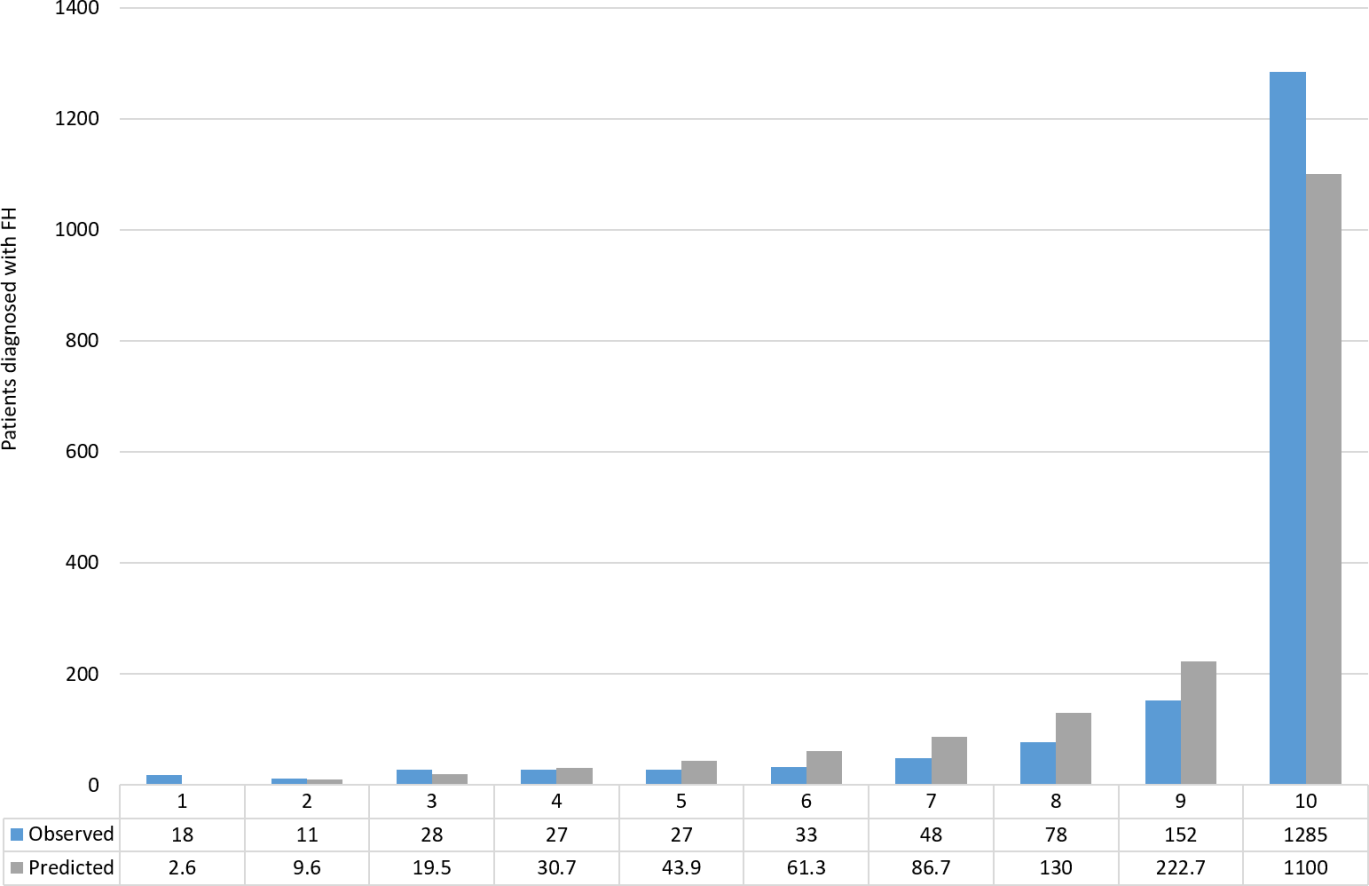
Calibration of observed versus predicted cases of familial hypercholesterolaemia (FH) in the external validation cohort by deciles of predicted probability using the optimised FAMCAT model

Fitting cholesterol as a continuous variable and including of personal history of premature MI and personal history of PVD, increased model discrimination by 5% (AUC = 0.894, 95% CI = 0.885 to 0.903) when compared to the validation model in the RCGP cohort. The optimised model showed good calibration across all deciles between observed and predicted cases (Figure 3). There was an expected sharp increase in observed and predicted cases in the highest decile of predicted probability where 1285 cases were observed and 1100 cases were predicted.

Using the same threshold corresponding to the top decile (10^th^) of predicted probability, a probability cut-off of ^1^/_250_ or 0.004, the optimised FAMCAT model achieved the following: a sensitivity of 69.4% (95% CI = 67.2% to 71.6%) and specificity of 92.8% (92.8% to 92.9%) with a corresponding positive predictive value of 1.58% (95% CI = 1.49% to 1.67%) and a negative predictive value of 100%. The optimised FAMCAT model improved specificity by 14.4% from the standard FAMCAT model.

## Discussion

### Summary

In this study, the FAMCAT algorithm has been validated in a separate cohort of over a million patients and has maintained high discriminatory accuracy. This algorithm also showed superior performance compared with recomended approaches in UK guidelines for case finding. It has also been demonstrated that the predictive accuracy of the FAMCAT algorithm can be further improved by incorporating personal history of premature MI and PVD, and fitting cholesterol levels as a continuous variable.

### Strengths and limitations

The study has a number of strengths, especially the large, population-based sample and a long duration of follow-up, to validate and optimise an algorithm that identifies patients with the highest probability of existing FH. The RCGP RSC data are nationally representative of the UK primary care patient population, and developed as a national disease and morbidity surveillance network. Given this purpose, disease coding and clinical measurements are better captured compared with other sources.^16^ For instance, the proportion of patients with family history of MI recorded (5.7% in men; 6.7% in women) is higher compared with the UK’s CPRD database (3.2% for both men and women).^13^

It is acknowledged that the study has limitations. The diagnosis of FH in the patient’s EHRs is based on the clinical phenotype, specifically those meeting clinical diagnostic criteria following specialist lipid assessment, which may or may not be confirmed by genetic testing. However, management of these patients to improve cardiovascular risk, will nevertheless be based on clinical phenotype. In UK national guidelines, the key role for genetic testing is to activate cascading testing to identify affected relatives by specialist care. The diagnosis is based on coded records rather than following an adjudication process, which would not be feasible in such a large cohort of patients. The use of unadjudicated diagnosis coded in records by clinicians, is widely adopted in major clinical epidemiological research.^23,24^

### Comparison with existing literature

FAMCAT is the first FH identification algorithm developed for use in the primary care setting. Other tools, developed to improve identification of FH in primary care, have incorporated DLCN criteria. This includes, the tool developed in the SEARCH Study,^25^ TARB-EX based on DLCN and correction for LDL-C,^26^ and the Caning Tool, an electronic extraction tool designed for primary care EHRs based on DLCN.^27^ The authors' previous study^28^ and current study show FAMCAT has significantly better predictive accuracy for clinical case finding than any of these approaches, including MEDPED, in very large primary care populations. The higher performance compared with recommended approaches is owing to it being developed directly from primary care EHRs. The nature of recording in routine EHRs has its limitations, hence, the application of very specific DLCN criteria developed outside primary care setting may not capture the distinict characteristics of individuals who may be at risk. Also, the use of blunt categorisations, such as total cholesterol and family history, or lipid levels alone, capture too many individuals who do not have FH.

### Implications for research and practice

The current and previous^28^ external validations of FAMCAT in most general practice systems now show it can be confidently applied across UK primary care to identify people with possible FH. As with all available approaches, FAMCAT will not identify everyone with FH in the general population. Rather, it offers an accurate and practical approach to case-find those patients most likely to have FH, so they can be referred for specialist assessment and definitive genetic diagnosis (or its exclusion). Other methods, such as child–parent screening or cascade testing in secondary care, could further improve identification of FH. The authors' further research is exploring using machine-learning (ML) to identify FH in primary care; alongside similar work using secondary care data.^29^

For clinical practice, the FAMCAT algorithm has been integrated into some GP computer systems as an automated case-finding tool: https://www.nottingham.ac.uk/primis/tools/qi-tools/familial-hypercholesterolaemia .aspx. Although this is available for UK practice, the FAMCAT variables are all routinely recorded so the tool could be developed for wider use internationally. A web-based FAMCAT online risk calculator is also now available: https://prism-uon.shinyapps.io/FAMCAT/.

In conclusion, this study confirms FAMCAT performs better than other recommended approaches to case finding for FH using Simon Broome criteria, the DLCN criteria, or very high cholesterol levels. Use of FAMCAT in general practice will identify those patients with possible FH most likely to need referral for specialist diagnosis, and greater intervention to reduce risk of premature heart disease.
